# Cumulative incidence and risk factors for limber tail in the Dogslife labrador retriever cohort

**DOI:** 10.1136/vr.103729

**Published:** 2016-06-27

**Authors:** C. A. Pugh, B. M. de C. Bronsvoort, I. G. Handel, D. Querry, E. Rose, K. Summers, D. N. Clements

**Affiliations:** The Roslin Institute and Royal (Dick) School of Veterinary Studies, University of Edinburgh, Easter Bush, Edinburgh EH25 9RG, UK

**Keywords:** Dogs, Case-control studies, Epidemiology, Welfare

## Abstract

Limber tail is a condition that typically affects larger working breeds causing tail limpness and pain, resolving without veterinary intervention. It is poorly understood and the disease burden has not been well characterised. Data collected from owners of the Dogslife cohort of Labrador Retrievers have been used to describe incidents and a case–control study was undertaken to elucidate risk factors with 38 cases and 86 controls. The cumulative incidence of unexplained tail limpness was 9.7 per cent. Swimming is not a necessary precursor for limber tail, but it is a risk factor (OR=4.7) and working dogs were more susceptible than non-working dogs (OR=5.1). Higher latitudes were shown to be a risk factor for developing the condition and the case dogs were more related to each other than might be expected by chance. This suggests that dogs may have an underlying genetic predisposition to developing the condition. This study is the first, large-scale investigation of limber tail and the findings reveal an unexpectedly high illness burden. Anecdotally, accepted risk factors have been confirmed and the extent of their impact has been quantified. Identifying latitude and a potential underlying genetic predisposition suggests avenues for future work on this painful and distressing condition.

## Introduction

Limber tail in dogs is a condition characterised by a flaccid tail, often with a stiff tail base. There is no clear case definition for the condition and it is diagnosed on the basis of signalment (being reported in larger working breeds), the clinical signs and their rapid self-resolution, which excludes other likely causes of tail flaccidity and stiffness. Dogs often show signs of pain and distress when they develop limber tail, although these resolve with the other clinical signs.

Limber tail was initially described in 1997 in the *Veterinary Record* as “acute onset paralysis of the tail (frozen tail or limber tail)” with onset following “swimming in, or showering with, cold water” (Hewison 1997). Subsequent letters described similar cases (Jeffels 1997, Steiss 1997a, Wilkins 1997) and Wilkins was the first to mention that the signs included a ‘painful tailbase’. The consensus was that the signs typically followed exercising in cold water and that they resolved after a period of as much as 10 days.

The authors of a Norwegian study (Bredal and Thoresen 1999) suggested that limber tail was caused by myositis. This theory was consistent with the findings of a small clinical and pathological study of English Pointers (Steiss and others 1999) comparing four case dogs with three controls from the same kennels. All affected dogs had flaccidity of the tail, raised creatine kinase levels indicative of myopathy and histological evidence of coccygeal muscle damage.

In a wider report on muscle disorders in working dogs (Steiss 2002), limber tail was described as a condition characterised by a flaccid tail which either hung from the base or extended horizontally for a short distance before hanging. Dogs would typically recover spontaneously within a few days to two weeks and anecdotal evidence indicated that NSAIDs minimised distress. The author referred to previous studies (Steiss and Wright 1995, Steiss 1996, 1997b,c), which included a survey of 113 owners of over 3000 hunting dogs in the Southeastern USA. The findings from this study have been widely paraphrased online but were originally published in magazines that are now unavailable.

In the academic literature, the first prevalence estimate for the condition was reported in 2008 from a convenience survey of lameness and injury involving over 1300 working dogs across two hunting seasons in 2005–2007 in Great Britain (Houlton 2008). Just three of 613 Labrador Retrievers (LRs) and one of 66 Flat-Coat Retrievers were reported to suffer from ‘cold tail’ in the study, resulting in an estimated risk of 0.49 per cent (95 per cent CI 0.1 to 1.4 per cent) in this group of working LRs. Thus this condition, which will henceforth be referred to as limber tail, appeared to have a low prevalence. In addition to the observation that limber tail is self-limiting, this might explain why there is a paucity of literature regarding the condition.

The Dogslife project is an ongoing cohort study of LRs in the UK (Clements and others 2013). Data are collected online directly from owners regarding the lifestyle, morphology and health of the dogs. The Dogslife database currently comprises information about over 6300 dogs. As such, it is ideally suited to investigate the prevalence and risk factors of conditions that may not be presented to veterinarians, such as limber tail. The purpose of this study was to describe the incidents of limber tail reported to Dogslife and undertake a case–control study to elucidate the risk factors associated with the condition.

## Materials and methods

The study was approved by the Veterinary Ethical Review Committee of the University of Edinburgh.

Dogslife participants are asked to complete an online questionnaire every month until the dog is one year of age and then quarterly after that. A description of the lifestyle and morphological data collected in the first three and a half years of Dogslife is available in Pugh and others (2015b). In addition to lifestyle information, owners are asked to report whether their dog has had any of six clinical signs indicative of illness such as diarrhoea or lameness and then “Did [dog's name] have any other illnesses or problems?” [yes/no]. If they answer [yes], then a free text box appears for them to detail the issue. They are asked for the dates when the clinical sign started and ended and whether they took the dog to their veterinarian with the problem. The details of the illness section of the online questionnaire were reported by Pugh and others (2015a).

### Stage 1

Dog-specific data were extracted from the Dogslife database based on owner descriptions of tail problems. The descriptions had to include the word ‘tail’ and any of the following words ‘cold’, ‘dead’, ‘droopy’, ‘drop’, ‘limb’, ‘limber’, ‘limp’, ‘rudder’, ‘staved’, ‘stiff’, ‘stride’, ‘swim’ or ‘swimmers’. Where tail was found without the additional keywords, the full text was examined. For example, “She had a problem with her tail, she couldn’t wag it for 5 days” was included as a possible case. The details of these incidents were collated.

### Stage 2

A case–control study was undertaken whereby provisional controls were chosen from those that had spent at least as much time in the Dogslife project as the case dogs identified above but had no report of signs of limber tail. They were chosen at random from 479 members of the Dogslife cohort whose owners had previously supplied saliva samples for DNA analysis, as this would facilitate future investigations of potential genetic predispositions. The saliva samples were originally collected from owners who had completed the Dogslife questionnaire at least three times.

Contemporaneously collected data for morphology and lifestyle were available for all Dogslife dogs. Additionally, the owners of the dogs in the case–control study were contacted and asked to complete one of two tail-specific questionnaires (see online supplementary materials 1 and 2). The questionnaires asked about swimming, which is not addressed in the online Dogslife questionnaire. In addition, information was collected about tail-related signs and about perceived pain levels and the impact on quality of life of potential limber tail incidents. In accordance with Steiss and others (1999), case and control status was determined using the following case definition: the dog had some degree of tail flaccidity but no report of tail injury or other cause. This was ascertained by asking about the following five signs relating to the tail:

1. It looks abnormally limp at the end
2. It looks abnormally limp along the entire length
3. It looks abnormally stiff at the base (near the body)
4. The hair on the top of it stands on end
5. It appears painful for no reason

10.1136/vetrec-2016-103729.supp1 Supplementary material 1

10.1136/vetrec-2016-103729.supp2 Supplementary material 2

All analyses were performed using R 3.2.2 (R Core Team. R: A language and environment for statistical computing. 2013. www.r-project.org/). The age, sex, coat colour, neuter status, household type, postcode location as country within the UK, owner smoking status, dog purpose and swimming activity were compared between the cases and controls using Fisher's exact tests (CIs were calculated using the *exact2×2* package (Fay 2010)). Using postcode locations to generate latitude and longitude coordinates as dependent variables, binary logistic regression models were used to compare case and control status as the response variable. Similarly, height, weight and exercise as reported to Dogslife (all scaled using R's scale function) were modelled as independent variables but in these instances, dog ID was included as a random effect to take into account repeated measures using the *lme4* package (Bates and others 2014). Risks and incidence rates are presented with exact binomial CIs.

In order to assess whether there was a difference in the relatedness of the cases and controls, permutation testing was undertaken whereby 10,000 samples the same size as the number of cases and controls were chosen at random from all Dogslife dogs and the number of different sires and dams contributing to these samples was determined. The dogs that contributed more than one offspring were weighted and averaged such that if N dogs were chosen at random and, of the sires or dams, one contributed three offspring, two contributed two and 31 contributed one, the value would equal 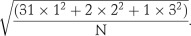


These values were treated as a distribution and compared with the number of sires and dams that contributed to the cases and controls.

## Results

Between July 2010 and October 2015, there were 53 possible incidents of limber tail reported to Dogslife, associated with 43 dogs. There were approximately 6000 Dogslife dogs enrolled in the study at this time. This would give a conservative cumulative incidence of 0.7 per cent (assuming owners of all dogs were reporting and all dogs were old enough to experience limber tail). This is similar to the previous value of 0.49 per cent (Houlton 2008). One dog had four reported episodes of limber tail, one had three, five had two and 36 had one. The mean age of the dogs at the first report was 2.13 years (95 per cent CI 1.75 to 2.50 years), median=1.64 years, range 8.6 months–5.0 years.

The owners described the incidents in 31 different ways and they can be summarised as follows: limp or limber tail (24), swim or swimmer's tail (3), frozen tail (3), cold tail (2), any combination of limber, swim, frozen or cold tail (9) and other (11). Of the owners who reported more than one incident, only one used the same phrasing for all incidents. Of all 53 incidents, only 11 prompted a veterinary visit and these 11 visits all related to different dogs. By implication, investigations using data collected at veterinary practices would miss 74 per cent of dogs exhibiting signs of the condition (95 per cent CI 58.8 to 86.5 per cent).

**FIG 1:**
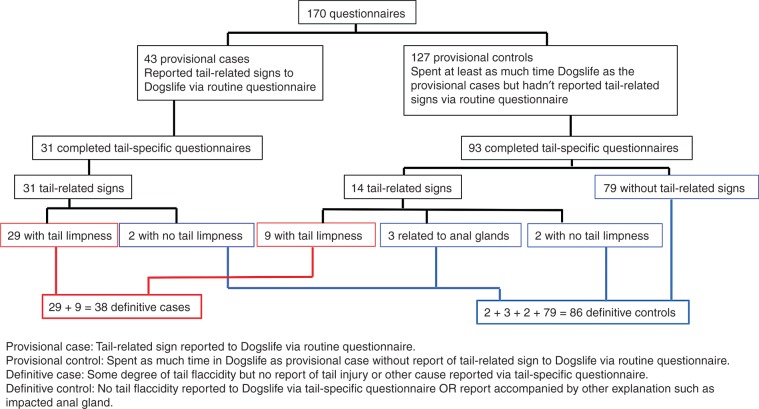
Description of provisional and definitive case and control status of the participating dogs

Between June and October 2015, the Dogslife project administrator attempted to contact 170 owners to ask them to complete a tail health questionnaire. The number of responses and how the dogs were categorised as cases or controls are shown in Fig 1. Questionnaires were returned by 124 owners (72.9 per cent). The questionnaires related to 31 of the 43 dogs provisionally identified as cases via routine reporting to Dogslife and 93 provisional controls. Responses to the detailed questionnaire indicated that two of the 31 provisional cases had no tail limpness so were excluded. The 93 provisional controls had a minimum age of 2.0 years and, in total, their lifetimes comprised 419 years at risk. Fourteen of them had one or more of the five signs that were identified as possibly indicative of limber tail. Of these 14, three owners reported that they suspected the signs (stiff base: two dogs, limp length: two dogs) were related to impacted anal glands and an additional two dogs had signs that did not include tail limpness. On this basis, nine of the provisional controls became cases. Although nine dogs were not identified as affected with limber tail by routine reporting, this was not unexpected as previous validation indicated under-reporting of illnesses to Dogslife (Pugh and others 2015c). The cumulative incidence of limber tail in these 93 dogs chosen randomly from a subset of the Dogslife cohort was 9.7 per cent (95 per cent CI 4.5 to 17.6 per cent), which is considerably higher than any previously reported estimate.

**FIG 2:**
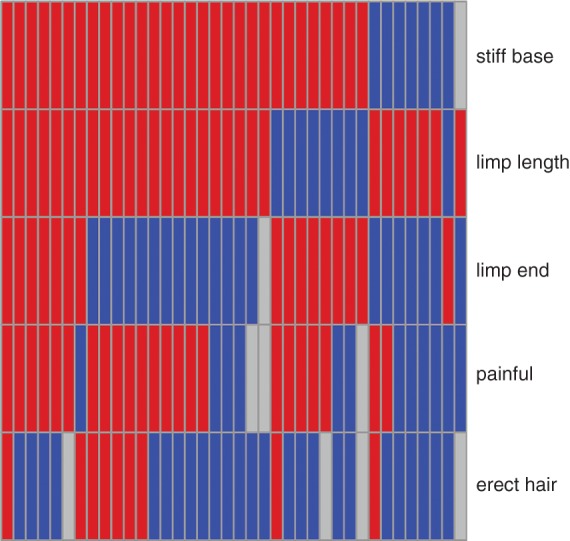
Tail-related signs seen during limber tail incidents. Each column relates to one dog. Owner answers were yes (red), no (blue) and unsure (grey)

For the 38 cases for which questionnaires were completed, the signs reported by owners are shown in Fig 2. It should be noted that 11 of the 12 owners who answered no to the pain question and two of the three who were unsure then gave non-zero answers on the pain scale. The mean pain score was 6.0 out of 10 (95 per cent CI 4.4 to 7.5) and in terms of quality of life where 0 referred to no impact and 10 implied the worst possible quality of life, the mean score was 4.1 (95 per cent CI 2.6 to 5.8). For both pain and quality of life, the reported range was 0–10. There was a strong correlation between pain and quality of life scores (Pearson's correlation coefficient=0.72).

Twenty-two of the dogs had a single incident, eight had two incidents, five had three, two had four and one dog was reported to have had approximately 30 incidents of limber tail. In terms of duration, there was considerable variation, both between dogs and between incidents for individual dogs. In all four cases where the owner gave different durations for different episodes, the later episodes were shorter. Overall, the shortest episode lasted just a few hours and the longest was reported to last about 10 days. If just the first incident for each dog was considered and ‘a few days’ was taken as three days, ‘a week or more’ was taken as eight days and a few hours was taken as three hours, then the mean duration was 3.5 days (95 per cent CI 2.9 to 4.2 days).

**FIG 3:**
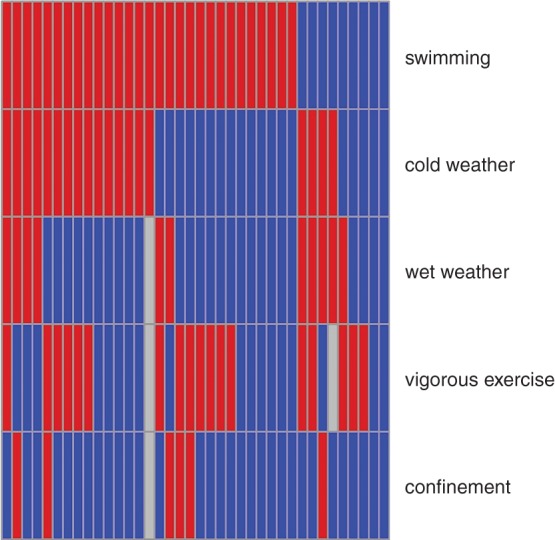
Diagram showing co-occurrence of precursors to limber tail. Each column relates to one dog. Owner answers were yes (red), no (blue) and unsure (grey)

Owners reported that the incidents followed: swimming (yes 29, no 9); exposure to cold weather (yes 19, no 19); vigorous exercise (yes 18, no 18, unsure 2); exposure to wet weather (yes 11, no 26, unsure 1) and/or confinement (crate 5, car 1, no 31, unsure 1). Fig 3, which is dominated by swimming and cold weather, shows how the precursors co-occurred. The owners of two case dogs could not recall their dog undertaking any of the five activities. To paraphrase, they suggested that limber tail had followed ‘over-excited wagging’ and ‘suspected banged tail when wagging vigorously’.

While it was clear that swimming was not a necessary precursor to limber tail, it still appeared to be a risk factor. When the swimming habits of the cases (36 swimmers, 2 non-swimmers) and controls (68 swimmers, 18 non-swimmers) were compared, the OR of a case swimming compared with a control was 4.7 (95 per cent CI 1.1 to 29.9), P=0.03.

Data were extracted from the Dogslife database relating to the cases and controls assigned according to the questionnaire as discussed above. The cases comprised 18 females and 20 males compared with 40 female and 46 male controls. The controls were slightly older than the cases, which was expected as they were chosen to have spent at least as much time in the Dogslife project as the cases. Otherwise, the cases and controls had similar distributions of neuter status, coat colour, height, weight, exercise levels, household type (eg ‘retired’) and whether anyone in the household smoked (see online supplementary Tables S1 and S2).

10.1136/vetrec-2016-103729.supp3 Supplementary tables

By contrast, there were a disproportionately high number of working dogs among the cases. The cases and controls were described, respectively, as household pets (32 and 82), working dogs (5 and 3), gundog and pet (1 and 0) and co-counsellor (0 and 1). The OR of a case being a working dog (including the dog that was a gundog and pet) compared with not being a working dog was 5.1 (95 per cent CI 1.1 to 24.9; Fisher's exact test, P=0.02). It also appeared that there was a geographical association. Using the postcode of their household, the dogs came from England (28 and 72), Scotland (9 and 12), Wales (1 and 0) and Northern Ireland (0 and 2) for cases and controls, respectively. The results of a binary logistic regression model indicated that the cases were disproportionately from higher latitudes with an OR of 1.47 (95 per cent CI 1.12 to 1.98) for each unit increase in latitude (regression parameters given in online supplementary Table S1).

**FIG 4:**
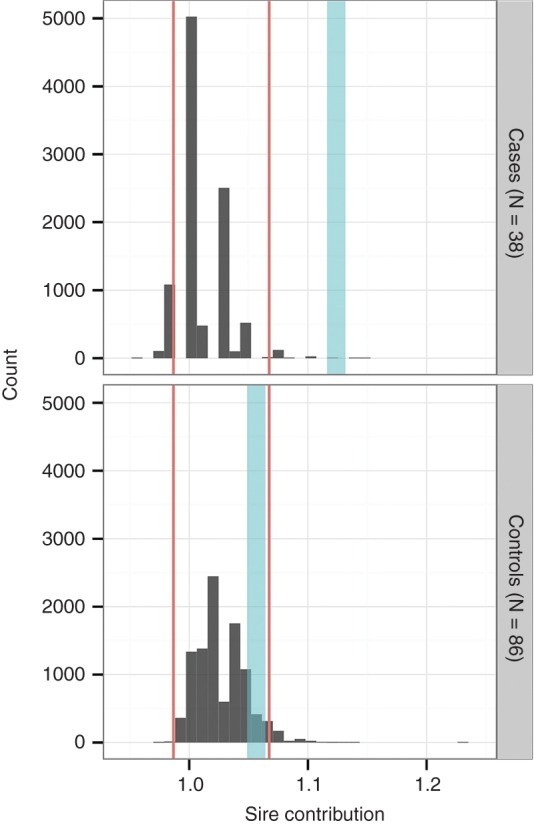
The distribution of 10,000 sire contributions generated randomly with percentiles (2.5 per cent and 97.5 per cent) are shown in red and the contributions of sires from the groups of cases and controls are shown in blue

The results of permutation testing indicated that, in terms of sires, the cases were more related than the controls and more related than would be expected from random selection from the cohort. Fig 4 shows the distribution of sire contributions for randomly generated samples of the same number as the cases and controls. The 2.5 per cent and 97.5 per cent percentiles are shown in red for each distribution and the contributions of sires to the cases and controls are shown in blue. The contribution of sires to the case dogs lies at the extreme right of the distribution (well beyond the 97.5th centile) and is significantly different from the randomly sampled distribution (P=0.0002).

## Discussion

This case–control study demonstrates the utility of owner-reported information regarding illnesses that are often not presented to veterinarians. It provides more information about a condition that has been underestimated and is poorly understood. Swimming has been confirmed as a risk factor but, despite prior suggestions that limber tail is caused by swimming (Hewison 1997, Jeffels 1997, Wilkins 1997, Houlton 2008), one-quarter of affected dogs did not swim prior to the onset of signs. In addition, working dogs have been shown to be more likely to suffer from the condition.

Increasing latitude has been newly identified as a risk factor for limber tail. The condition has also been called ‘cold tail’ (Houlton 2008) and given that temperature is intrinsically linked with latitude, this is the first evidence to support the anecdotal accounts of association with colder temperatures.

Under-reporting of limber tail was confirmed in the cohort. Indeed, an initial cumulative incidence of 0.7 per cent was rapidly superseded by the cumulative incidence in the provisional controls of 9.7 per cent. It is possible that the 93 provisional controls that chose to answer the questionnaire were disproportionately likely to have suffered from tail-related signs, making this an over-estimate. However, even if the 34 non-responders are treated as controls, that still leaves a cumulative incidence of 7.1 per cent (9/127). The potential controls were considerably older than the wider cohort that contributed to the 0.7 per cent cumulative incidence, which included dogs of just a few months of age but 9.7 per cent (or even 7.1 per cent) was an unexpectedly high value. This may be due to ambiguity regarding diagnosis; as mentioned previously, limber tail is a diagnosis of exclusion. Furthermore, given that there appears to be an environmental component to limber tail, this new estimate may also be an under-estimate in terms of susceptibility because many more dogs may never be exposed to triggering environmental risk factors.

While it is surprising that so many of the controls were actually cases that owners had not mentioned previously, routine reporting to Dogslife identified more potential cases than were presented to veterinarians. The study reveals a previously unsuspected burden of the condition. This fits with the phenomenon known as the ‘symptom iceberg’ described in people (Last and Adelaide 1963) whereby symptoms of illness presented to doctors are just a small proportion of all symptoms of illness suffered.

Owner perception of the pain caused and impact on the dog's quality of life was quantified with a relatively high mean of 6.3 out of 10 for pain levels. The subjectivity of both measures makes the results interesting because while only two owners reported a zero pain score, five owners, including two owners who gave pain scores greater than seven, gave zero for the impact on quality of life. The difference was not obviously explained by the reported number of episodes and duration of signs. There was a strong correlation between the two measures, but some owners perceived high pain levels to have no impact on the dog's quality of life. This finding is similar to previous work indicating that, when considering quality of life, owners do not consider just pain but also the dog's ability to perform its normal daily activities (Brown and others 2013).

Of all 53 incidents, only 11 prompted a veterinary visit and all 11 visits related to different dogs. The perception of some owners that the condition has minimal impact on quality of life may be one underlying reason for not seeking veterinary advice. It may also reflect owner familiarity with the condition. Once encountered, an owner would likely recognise future incidents and be aware that the signs will resolve without intervention. Nevertheless, by implication, investigations using data collected at veterinary practices would miss nearly three-quarters of dogs exhibiting signs consistent with the condition.

This study has shown that aspects of lifestyle are associated with limber tail incidence but no associations were found with dog height, weight, coat colour, household type, owner smoking status and reported exercise levels in this cohort. Initial comparisons of the pedigrees of the cases and controls indicate that there may be an underlying genetic risk factor, which should be investigated further to give a better overall picture of disease aetiology. Given the environmental risks, the data suggest that a gene–environment interaction is responsible for mediating the disease.

The study demonstrates that it is possible to collect condition-specific information from Dogslife contributors via a specific questionnaire. The data were not collected contemporaneously so they may be subject to recall bias but the extra information collated was invaluable for better characterising the condition and the risks for its development. It enabled tail-related signs associated with limber tail to be described.

The authors highlight the need for further such investigations. It is hoped that future work by the Dogslife project team will enable the identification of genes associated with limber tail. Such an investigation would be complicated by environmental risk factors because it will be difficult to determine whether dogs that had no report of limber tail were truly not susceptible to the condition or had simply not been exposed to factors which might lead to the development of signs. Nevertheless, it appears that the extent of limber tail has been underestimated and if a genetic predisposition could be identified, breeding stock could be selected to reduce the prevalence of the condition, particularly in working dogs.
