# Supplementary material for: Cumulative incidence and risk factors for limber tail in the Dogslife labrador retriever cohort
Source: Vet Rec. 2016 Jun 27;179(11):275. doi: 10.1136/vr.103729 (PMC5036231; doi:10.1136/vr.103729)
Supplement: Supplementary material 1 [file vetrec-2016-103729supp_material1.pdf]

Limber Tail Questionnaire

Thank you for participating in the Dogslife project. We are currently investigating factors that cause limber tail (and also known as cold tail, swimmers tail, frozen tail, rudder tail or limp tail) in Labrador Retrievers. We would like to find out more about the condition. As your dog is reported to have had this condition we would be very grateful if you could detail the following information about your dog, and return the questionnaire to us by e-mail (info@dogslife.ac.uk) or in the postal envelope provided. Thank you for your time and help.

(* = please circle the most appropriate answer)

1. **How many separate episodes of limber tail** has your dog had? …….

2. **Do the episodes follow?***

   a. Swimming? *Yes / No*
   b. Cold Weather? *Yes / No*
   c. Wet weather? *Yes / No*
   d. Vigorous exercise? *Yes / No*
   e. Resting in a confined area (e.g. dog crate) *Yes / No*
   f. Anything else? ……………………………………………………

3. **What does your dog's tail look like when the episodes occur**?*

   a. Limp at the end *Yes / No*
   b. Limp along the entire length *Yes / No*
   c. Stiff at the base (near the body) *Yes / No*
   d. The hair on the top of it stands on end *Yes / No*
   e. It appears painful for no reason *Yes / No*

4. On average, **how long does the episode last**?**An hour / a few hours / a day / a few days / a week or more*

5. **Can you avoid the episodes occurring**?* *Yes / No*

   If yes, how? ……

6. On a scale of 0 to 10**, how painful would you say each episode is?**

   (0 = not painful, 10 = could not be more painful) …….

7. On a scale of 0 to 10, **how much does this condition affect your dog's quality of life?**

   (0 = does not affect my dogs quality of life, 10 = my dog's quality of life could not be worse, because of this condition)? …….

8. **Is there anything else you would like to tell us about this condition**?

   …….......

9. **Does your dog go swimming**?* *Yes / No*

   If YES, **when** (all the year or just in summer), **how often** (daily or occasionally) and **where** (sea, canals, rivers etc.) does your dog go swimming?

   ……..

Thank you for this extra information about your dog; this is a tremendous help to the Dogslife project.
