# Supplementary material for: Cumulative incidence and risk factors for limber tail in the Dogslife labrador retriever cohort
Source: Vet Rec. 2016 Jun 27;179(11):275. doi: 10.1136/vr.103729 (PMC5036231; doi:10.1136/vr.103729)
Supplement: Supplementary material 2 [file vetrec-2016-103729supp_material2.pdf]

Tail Health Questionnaire

Thank you for participating in the Dogslife project. We are currently investigating factors that cause limber tail (and also known as cold tail, swimmers tail, frozen tail, rudder tail or limp tail) in Labrador Retrievers - to find out more about the condition you can read our previous newsletter article about it here: http://www.dogslife.ac.uk/newsletter/view/17#tail. As your dog has never been reported to Dogslife as having had this condition, we wanted to double-check some information with you so that we can compare your dog to others who are affected. We would be very grateful if you could complete the questionnaire and return it to us by e-mail (info@dogslife.ac.uk) or in the postal envelope provided. Thank you for your time and help.

(* = please circle the most appropriate answer)

1. **Does your dog go swimming**?* *Yes / No*

   If YES, **when** (e.g. all the year or just in summer), **how often** (e.g. daily or occasionally) and **where** (e.g. sea, canals, rivers etc.) does your dog go swimming?

   ……….

2. **Has your dog ever had any episodes where they display any of the following signs with their tail**?*

   a. It looks abnormally limp at the end *Yes / No*
   b. It looks abnormally limp along the entire length *Yes / No*
   c. It looks abnormally stiff at the base (near the body) *Yes / No*
   d. The hair on the top of it stands on end *Yes / No*
   e. It appears painful for no reason *Yes / No*

If you answered NO to ALL of the responses in Question 2, you have completed the questionnaire
If you answered YES to ANY of the responses in Question 2, please answer all the following questions;

3. On average, **how long does an episode last**?* *An hour / a few hours / a day / a few days / a week*

4. **Do the episodes follow?***

   a. Swimming? *Yes / No*
   b. Cold Weather? *Yes / No*
   c. Wet weather? *Yes / No*
   d. Vigorous exercise? *Yes / No*
   e. Resting in a confined area (e.g. dog crate) *Yes / No*
   f. Anything else? ………………………………………………………

5. **Can you avoid the episodes occurring**?* *Yes / No*

   If yes, how? ………..

6. On a scale of 0 to 10**, how painful would you say each episode is?**

   (0 = not painful, 10 = could not be more painful) …….

7. On a scale of 0 to 10, **how much does this condition affect your dog's quality of life?**

   (0 = does not affect my dogs quality of life, 10 = my dog's quality of life could not be worse, because of this condition)? …….

8. **Is there anything else you would like to tell us about this condition**?

   ……….

Thank you for this extra information about your dog; this is a tremendous help to the Dogslife project.

The Roslin Institute, a world class research centre, is incorporated with the Royal (Dick) School of Veterinary Studies, the University of Edinburgh
The Roslin Institute receives strategic funding from the BBSRC
The University of Edinburgh is a charitable body, registered in Scotland, with registration number SC005336
