# Supplementary material for: Cumulative incidence and risk factors for limber tail in the Dogslife labrador retriever cohort
Source: Vet Rec. 2016 Jun 27;179(11):275. doi: 10.1136/vr.103729 (PMC5036231; doi:10.1136/vr.103729)
Supplement: Supplementary tables [file vetrec-2016-103729supp_tables.pdf]

Supplementary Table 1: Results of logistic regression analyses

Variable	Estimate	Std Error	z-value	P value
Weight [ab]	-0.074	0.865	-0.086	0.932
Height [ab]	0.047	0.82	0.058	0.954
Daily time spent exercising [b]	0.094	0.85	0.111	0.912
Latitude [c]	0.386	0.14	2.668	**0.008**
Longitude [c]	0.285	0.15	1.909	0.056

[a] Sex of the dog was included as a potentially relevant additional fixed effect in these models
[b] These measures were scaled using the *scale* function in R and the dog ID was included as a random effect in order to deal with repeated measurements.
[c] Combined model including both latitude and longitude

Supplementary Table 2: Comparison of characteristics between cases and controls with results of Fisher's exact tests

Coat Colour	Black	Yellow	Chocolate	Fox Red	Other	Fisher's Exact P-Value
Cases	21	8	6	1	2	
Controls	46	21	17	2	0	0.337
Neutering	Neutered	Entire				
Cases	27	11				
Controls	67	19				0.496
Owner smoking status	Non-Smoker	Non-Smoker	Unknown			
Case	34	3	1			
Control	73	12	1			0.439
Household type	More than one adult	Family	Retired	Single adult	Unknown	
Case	17	13	5	2	1	
Control	37	29	11	8	1	0.929
